# Heterogeneous Downregulation of Angiotensin II AT1-A and AT1-B Receptors in Arterioles in STZ-Induced Diabetic Rat Kidneys

**DOI:** 10.1155/2014/947506

**Published:** 2014-01-21

**Authors:** Zsolt Razga, Gabor Kovacs, Nikolett Bódi, Petra Talapka, Jens Randel Nyengaard

**Affiliations:** ^1^Department of Pathology, University of Szeged, Hungary; ^2^Department of Pediatrics, University of Szeged, Állomás Utca 2, Szeged 6725, Hungary; ^3^Department of Physiology, Anatomy and Neuroscience, University of Szeged, Hungary; ^4^Electron Microscopy and Stereology Research Laboratory, University of Aarhus, Denmark

## Abstract

*Introduction.* The renin granulation of kidney arterioles is enhanced in diabetes despite the fact that the level of angiotensin II in the diabetic kidney is elevated. Therefore, the number of angiotensin II AT1-A and AT1-B receptors in afferent and efferent arteriole's renin-positive and renin-negative smooth muscle cells (SMC) was estimated. *Method.* Immunohistochemistry at the electron microscopic level was combined with 3D stereological sampling techniques. *Results. *In diabetes the enhanced downregulation of AT1-B receptors in the renin-positive than in the renin-negative SMCs in both arterioles was resulted: the significant difference in the number of AT1 (AT1-A + AT1-B) receptors between the two types of SMCs in the normal rats was further increased in diabetes and in contrast with the significant difference observed between the afferent and efferent arterioles in the normal animals, there was no such difference in diabetes. *Conclusions*. The enhanced downregulation of the AT1-B receptors in the renin-negative SMCs in the efferent arterioles demonstrates that the regulation of the glomerular filtration rate by the pre- and postglomerular arterioles is changed in diabetes. The enhanced downregulation of the AT1-B receptors in the renin-positive SMCs in the arterioles may result in an enhanced level of renin granulation in the arterioles.

## 1. Introduction

The juxtaglomerular apparatus (JGA) is a morphological complex which regulates the glomerular filtration rate. All of its elements are controlled by the renin angiotensin system (RAS) via the angiotensin II receptors. The activity of the RAS on JGA is expressed in the number of angiotensin II receptors, such as the AT1-A, AT1-B and AT2 receptors. The effect of the angiotensin II on the arteriolar tone is related to the AT1-A and AT1-B, receptors [1]. In normal rat kidney arterioles, the number of AT1-A receptors is downregulated in the renin-positive smooth muscle cells (SMCs) as compared with the renin-negative SMCs [2]. There is a significant difference between the afferent and efferent arterioles in the number of AT1-B receptors, which are upregulated on the SMC surface on the efferent arterioles [2].

Via the negative feedback, angiotensin II inhibits the transdifferentiation between the renin-negative SMC and renin-positive SMC. In response to treatment with an angiotensin II blocker, such as candesartan, the extent of renin granulation of the afferent arterioles is increased [3, 4]. The level of angiotensin II in the diabetic kidney is significantly higher than the normal level, while the level of renin granulation of the afferent arterioles is increased [5, 6]. Thus, the negative feedback from renin synthesis should be downregulated (through an unknown mechanism) because the extent of renin granulation of the afferent arterioles is higher than normal [7]. Our theory suggests that the number of AT1 receptors is downregulated not only in the SMC, but also to an extreme degree in the renin-granulated SMCs in diabetes. We have estimated the numbers of angiotensin II AT1-A and AT1-B receptors on the surface of renin-positive and renin-negative SMCs. Since the arteriolar tone is controlled by angiotensin II via the AT1 receptors [1], the AT1 receptor subtypes in diabetic rat kidneys were estimated on the afferent and efferent arterioles separately.

## 2. Materials and Method

### 2.1. Animals with STZ-Induced Diabetes

Streptozotocin (Sigma, USA) was administered intraperitoneally in dose of 60 mg/kg to adult male Wistar rats (300–400 g; *n* = 5). After 48 h, the blood sugar level was measured; those animals where the blood sugar level exceeded 18 mM were regarded as diabetic. The blood sugar level and the weight of the animals were followed over a period of 10 weeks, after which the rats were killed.

### 2.2. The Measurement of Angiotensin II Level

The sample collections (blood: phlebotomy from Vena cava posterior-vacutainer EDTA tube; kidney: after laparotomy kidneys were excised, purified from adipose tissue) at the end of the streptozotocin treatment were carried out. It was used the Uscn Life Science Inc. ELISA kit (Cat.no.US-E90005Ra) to determine the level of angiotensin II from bloods and kidney tissue samples, which has high sensitivity for detection of Angiotensin II. The preparation of tissue and blood was strictly followed in accordance with the user manual of the kit.

### 2.3. Immunohistochemistry

For immunohistochemical purposes, the same protocol was performed as described earlier [2]. Sections placed on glass slides were washed in PBS (pH = 7.4) for 10 min and then incubated in normal goat serum 1 : 100 (EMS, USA) in PBS (pH = 7.4) for 20 min. The primary antibody against the AT1-A or AT1-B receptor of angiotensin II (rabbit polyclonal, Advance Targeting Systems, San Diego, USA) was diluted 1 : 50 in PBS (pH = 7.4) and left overnight at 4°C in a wetting chamber. The sections were subsequently washed three times for 5 min each in PBS (pH = 7.4). 0.8 nm gold-conjugated goat-anti-rabbit IgG (Aurion Immunogold, EMS, USA) was diluted 1 : 100 in PBS (pH = 7.4), allowed to stand for 2 h, and then washed three times for 5 min each in PBS (pH = 7.4). The sections were fixed with 3% glutaraldehyde in PBS (pH = 7.4) and washed with distilled water for 5 min, followed by silver enhancement with Danscher solution (Aurion R-gent SE-EM, EMS, USA) for 75 min at 25°C, resulting in a particle size around 30 nm. Finally, the sections were washed three times for 5 min each with distilled water.

### 2.4. Embedding and Sectioning

The immunostained serial sections placed on glass slides were dehydrated with ordinary series of ethanol and acetone and infiltrated for 10 min with epoxy resin (TAAB 812). The sections were then capped with plastic capsules filled with clean resin. The semithin (0.4 *μ*m) and thin (70 ± 5 nm; mean ± SD) sections were prepared with an Ultracut S (Leica) ultramicrotome. The thin sections were stained with uranyl acetate and lead citrate.

*Stereology. *For sampling, the same protocol was applied as previously [8]. An afferent arteriole was defined as a preglomerular arteriole from the glomerulus to the first branching. An efferent arteriole was defined as a postglomerular arteriole from the glomerulus to the capillary. Each arteriole was followed on series of semithin sections, and profiles appearing from the same arteriole were identified [9, 10]. The TEM micrographs were prepared with an Analysis software 3.1 module (Soft Image System GmbH, Münster) on a Philips CM10 electron microscope (FEI, Eindhoven) equipped with a MegaView II camera. The immunohistochemical signals of AT1-A or AT1-B receptor molecules were counted on the surface of arteriolar SMCs, renin-granulated cells, and endothelial cells of afferent and efferent arterioles. More details are to found in recent articles by Razga and Nyengaard [2, 11].

### 2.5. Statistics

For the comparison of differences between the two groups, Student's *t*-test was applied.

## 3. Results

### 3.1. Diabetic Animals

The STZ treatment was successful: the blood sugar level of the animals had increased 3-fold significantly by the end of 10 weeks (Table 1).

### 3.2. The Angiotensin II Level in Plasma and Kidney

There were no significant difference between the control and diabetic in the angiotensin level of plasma or kidney after the 10-week STZ treatment (Table 2).

### 3.3. Regulation of AT1-A and AT1-B Receptors

**Figure 1:**
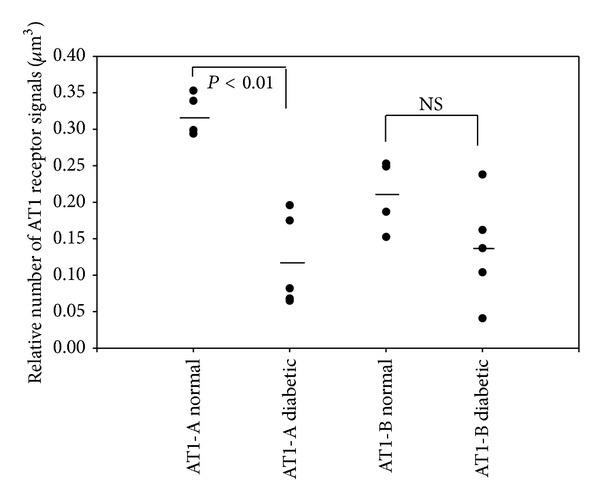
Downregulation of the AT1 receptor subtypes in the endothelia in the arterioles.

**Figure 2:**
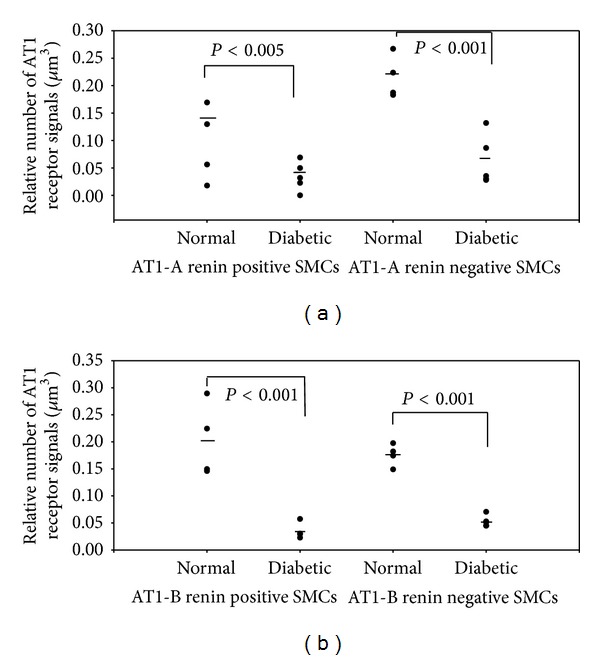
The angiotensin II AT1 receptors are downregulated in both renin-positive and renin-negative SMCs in arterioles.

**Figure 3:**
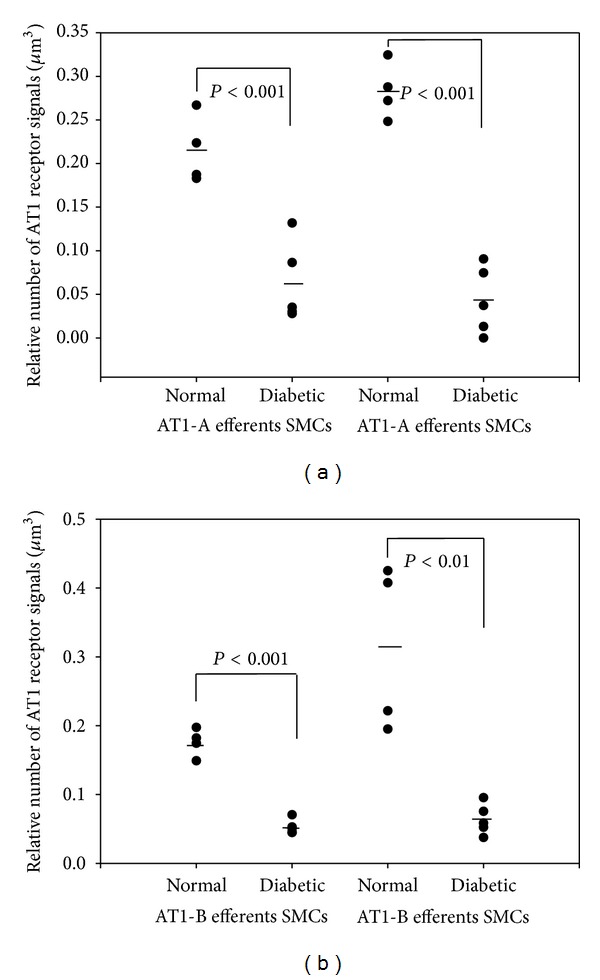
The angiotensin II AT1-B receptor is downregulated in the SMCs of the afferent and efferent arterioles.

**Figure 4:**
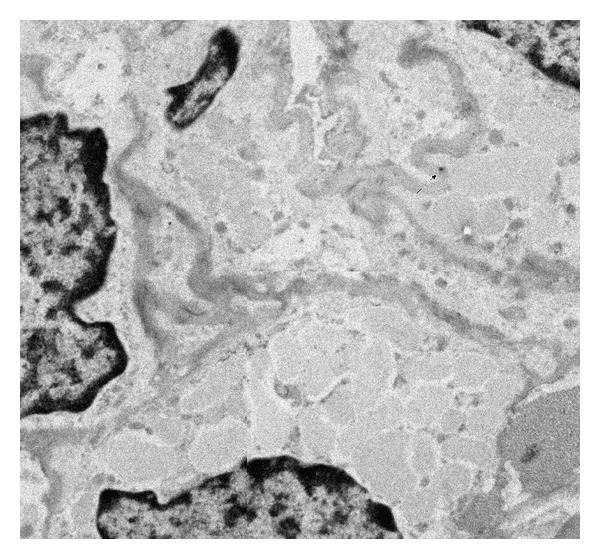
Immunohistochemical staining of AT1-A receptors on renin-positive SMCs of afferent arteriole in diabetes. There is less signal on cell surface than in normal. The original magnification of TEM micrograph was 13500x (1972 × 1828 pixel).

**Figure 5:**
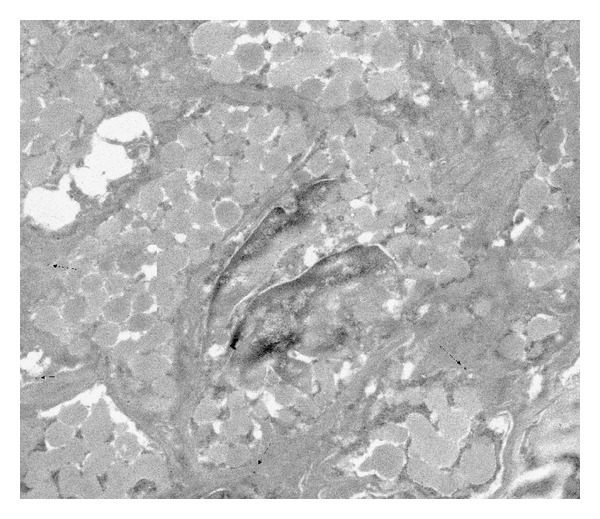
Immunohistochemical staining of AT1-A receptors on renin-positive SMCs of afferent arteriole in normal rats. There is more signal on cell surface than in diabetes. The original magnification of TEM micrograph was 13500x (2152 × 1844 pixel).

The downregulation of the AT1-A receptors in the endothelial cells of the arterioles differed from that of the AT1-B receptors (Figure 1). The AT1-A receptors were downregulated equally in the renin-positive (Figures 4 and 5) and renin-negative SMCs (Figure 2(a)), while in diabetes the significant difference in the number of AT1-A receptors between the renin-positive and renin-negative SMCs was unchanged (Table 3). The AT1-B receptors were downregulated more in the renin-positive than in renin-negative SMCs in both types of arterioles (Figure 2(b); Table 3). The significant difference in the number of AT1 (AT1-A + AT1-B) receptors between the two types of SMCs in the normal rats was further increased in diabetes (Table 3). The AT1-B receptors were downregulated more in the efferent than in the afferent arterioles (Figures 3(a) and 3(b)); in contrast with the significant difference observed between the afferent and efferent arterioles in the normal animals, there was no such difference in diabetes (Table 4).

## 4. Discussion

Our results demonstrated that AT1-A or AT1-B receptors of angiotensin II downregulated heterogeneously in different cells and arterioles, while the angiotensin II level in plasma and kidney does not show any significant differences after 10 weeks of STZ treatment. The angiotensin II level in kidney increased by STZ-induced diabetes 12–20 weeks [12]. In our project the evidence that the proceedings of angiotensin II receptors downregulation are independent from angiotensin II level was provided.

### 4.1. Specificity of Antibodies Labeled AT1-A and AT1-B Receptors

The selectivity of primary antibody against the AT1-A or AT1-B receptors of angiotensin II was guaranteed in earlier demonstration of estimating the AT1-A or AT1-B receptor of angiotensin II in afferent and efferent arterioles of kidney in normal rat [2]. In this study the results from the earlier job using the nonselective primary antibody against the subtypes of AT1 receptors (rabbit IgG; Santa Cruz Biotechnology, USA) by using the two primary antibodies selective to AT1-A or AT1-B receptors (rabbit polyclonal, Advance Targeting Systems, San Diego, USA) [2, 11] were reproduced.

### 4.2. The Effect of Insulin on the Regulation of the AT1 Receptors

Insulin causes an upregulation of the AT1 receptor gene expression in the vascular SMCs in cell cultures. The AT1 receptor upregulation results in an enhanced functional response of the vascular SMCs on stimulation with angiotensin II [13, 14]. In our experiment, 10 weeks after the onset of STZ-induced diabetes, the AT1 receptors (AT1-A and AT1-B) were downregulated in the afferent and efferent arterioles; this downregulation was extreme and heterogeneous. In normal cells, the number of AT1-A receptors on surfaces of renin-positive and renin-negative SMCs was earlier reported to be decreased by 27–40% [2, 11], while the downregulation in our diabetic rats in the different cells of the JGA range between 61 and 82% (Figures 1–3). This level of downregulation in diabetes is extreme compared to the normal level. It is heterogeneous because the AT1-A and AT1-B receptors are downregulated to different extents in the various cell types in the JGA. Differences were also found between the afferent and efferent arteriolar SMCs in the downregulation of the AT1-B receptor. One possible reason for the significant and heterogeneous downregulation of the AT1 receptors on the cells of the arterioles is the insulin level reduction caused by STZ treatment.

### 4.3. Regulation of AT1 Receptor Subtypes in the Afferent and Efferent Arterioles in Diabetic Kidneys

Angiotensin II regulates both the afferent and the efferent arteriolar tone via the AT1 receptors [1, 15]. The physiological data indicate that angiotensin II exerts a stronger contractility effect on the postglomerular than on the preglomerular arterioles in normal animals [16]. The mechanisms of these heterogeneous contractile effects were clarified by our earlier work, as the angiotensin II AT1 receptor is upregulated in the efferent arterioles as compared with the afferent arterioles, and this upregulation is specific for the AT1-B receptor [2, 11]. The angiotensin II level in the kidney is elevated in STZ-induced diabetes [5, 6, 12]. Our results have demonstrated that the AT1-A and AT1-B receptors are downregulated in the afferent and efferent arterioles in STZ-induced diabetic kidneys. This downregulation of the AT1-A and AT1-B receptors could compensate for the contractile effect on the arteriolar SMCs by a high level of angiotensin II in diabetic kidneys.

The efferent arteriolar contraction brought about by angiotensin II results in the opposite effect on the GFR to that caused by afferent arteriolar contraction [17]. In diabetes, the downregulation of the AT1 receptors on the efferent arterioles was enhanced in the AT1-B subtype, which changed to equal the significant difference in the total number of the AT1-B subtypes between the afferent and efferent arterioles of normal kidneys. This heterogeneous downregulation of the AT1-B receptor might decrease the efferent arteriolar effect of angiotensin II on the GFR.

### 4.4. Regulation of Renin Synthesis in STZ-Induced Diabetes

Renin synthesis is enhanced by angiotensin II via the AT1 receptors in the collecting ducts [16, 18]. An opposite effect of angiotensin II occurs in the afferent arterioles, where angiotensin II inhibits the transdifferentiation between the renin-negative SMCs and the renin-positive SMCs via the AT1 receptors. The receptor that mainly regulates this trans-differentiation is the AT1-A receptor [2].

In STZ-induced diabetic rats, the angiotensin II level in the kidney is higher than in normal rats [5, 6, 12]. Thus, the extent of renin synthesis in the collecting ducts should be increased and the level of renin granulation of the afferent arterioles should be decreased. The former holds true for diabetes: the main source of renin is the collecting duct [5, 6, 16], but the level of renin granulation in the afferent arterioles is higher in diabetes [7]. Our results have revealed that the AT1-A and AT1-B receptors were downregulated in both the renin-positive and renin-negative SMC surfaces. As concerns the AT1-A receptors the difference between the renin-positive and renin-negative SMCs is the same as in normal rats. However, the AT1-B receptors are downregulated more in the renin-positive than in the renin-negative SMC. The total number of AT1 (AT1-A + AT1-B) receptors is reduced in diabetes. One possible reason for the high level of renin granulation of the afferent arterioles with the high level of angiotensin II in diabetes might be the enhanced downregulation of the AT1-B receptors of the SMC.

## 5. Conclusions

In diabetes, the activity of the RAS is downregulated less in the endothelial cells than in the SMC. The enhanced downregulation of AT1-B in the renin-negative SMCs of the efferent arterioles demonstrates that the regulation of the glomerular filtration rate by the pre- and postglomerular arterioles is changed in diabetes. The enhanced downregulation of the AT1-B receptors in the renin-positive SMCs of the arterioles may result in an enhanced extent of renin granulation of the arterioles in response to high levels of angiotensin II in the diabetic kidney.

## Figures and Tables

**Table 1:** Changes in blood sugar level and weight following the STZ diabetic protocol.

			Animal weight (g)	Blood sugar level (mM/L)	
			Mean	Mean	SD
Control	Start	*n* = 4	354	6.7	0.43
	10 weeks	*n* = 4	457	5.53	0.86
Diabetic	Start	*n* = 5	360	6.25	0.49
	10 weeks	*n* = 5	318	20.25	9.68

**Table 2:** Angiotensin II level in plasma and kidney (pg/mL).

	*n*	Control	Diabetes	
		Mean (CV)	Mean (CV)	
Angiotensin II plasma level (pg/mL)	3	270 (0.56)	246 (0.29)	NS
Angiotensin II kidney level (pg/mL)	3	666 (0.02)	668 (0.04)	NS

**Table 3:** The relative numbers of angiotensin II AT1 receptor subtypes in the renin-negative and renin-positive SMCs in STZ-induced diabetic and normal rat kidney arterioles. The normal state in the number of AT1-B receptors between the renin-negative and renin-positive SMCs is changed to significantly different in diabetes, because of the enhanced downregulation of the AT1-B receptors in renin-positive SMCs.

		*n*	Renin-positive SMCs	Renin-negative SMCs	Difference	*P*
			Mean (CV)			
Angiotensin II AT1-A receptors in afferent arterioles cells surface (signals/um^3^)	Normal	4	0.133 (0.42)	0.215 (0.18)	38%	0.026
Angiotensin II AT1-B receptors in afferent arterioles cells surface (signals/um^3^)	Normal	4	0.203 (0.34)	0.176 (0.11)	13.3%	NS
Angiotensin II AT1-A receptors in afferent arterioles cells surface (signals/um^3^)	Diabetic	5	0.0345 (0.52)	0.058 (0.31)	41%	0.035
Angiotensin II AT1-B receptors in afferent arterioles cells surface (signals/um^3^)	Diabetic	5	0.033 (0.45)	0.055 (0.16)	40%	0.0096
Angiotensin II AT1-(A + B) receptors in afferent arterioles cells surface (signals/um^3^)	Normal	4	0.156 (0.27)	0.216 (0.18)	27.8%	0.043
Angiotensin II AT1-(A + B) receptors in afferent arterioles cells surface (signals/um^3^)	Diabetic	5	0.035 (0.30)	0.057 (0.38)	37.8%	0.041

**Table 4:** The relative numbers of angiotensin II AT1 receptor subtypes in SMCs in the afferent and efferent arterioles in STZ-induced diabetic and normal rat kidneys. The normal state in the number of AT1-B receptors between the afferent and efferent arteriole is changed to equal in diabetes, because of enhanced downregulation of AT1-B receptor in the efferent arteriole.

		*n*	Afferent arterioles	Efferent arterioles	Difference	*P*
			Mean (CV)		
Angiotensin II AT1-A receptors in SMCs surface (signals/um^3^)	Normal	4	0.215 (0.18)	0.283 (0.19)	24%	NS
Angiotensin II AT1-B receptors in SMCs surface (signals/um^3^)	Normal	4	0.176 (0.11)	0.312 (0.38)	44%	0.034
Angiotensin II AT1-A receptors in SMCs surface (signals/um^3^)	Diabetic	5	0.062 (0.73)	0.043 (0.9)	30%	NS
Angiotensin II AT1-B receptors in SMCs surface (signals/um^3^)	Diabetic	5	0.053 (0.19)	0.064 (0.34)	17%	NS
